# Effects of Hyperuricemia on Renal Function of Renal Transplant Recipients: A Systematic Review and Meta-Analysis of Cohort Studies

**DOI:** 10.1371/journal.pone.0039457

**Published:** 2012-06-22

**Authors:** Yan Huang, Yu-Lin Li, He Huang, Ling Wang, Wen-Ming Yuan, Jing Li

**Affiliations:** 1 Chinese Cochrane Center, West China Hospital, Sichuan University, Chengdu, People’s Republic of China; 2 The Second Clinical Medical College of Chengdu University of TCM, Chengdu University of TCM, Chengdu, People’s Republic of China; 3 Department of Cardiology, West China Hospital, Sichuan University, Chengdu, People’s Republic of China; 4 Department of Cardiology, The People’s Hospital of Mianyang, Mianyang, People’s Republic of China; 5 Department of Nephrology, The First Affiliated Hospital of Zhengzhou University, Zhengzhou University, Zhengzhou, People’s Republic of China; Mario Negri Institute for Pharmacological Research and Azienda Ospedaliera Ospedali Riuniti di Bergamo, Italy

## Abstract

**Background:**

Hyperuricemia is an independent risk factor of nephropathy, but its role in renal transplant recipients (RTRs) is controversial.

**Methods:**

Based on the methods of Cochrane systematic reviews, we searched MEDLINE (1948–2011.6), EMBASE (1956–2011.6), CBM (Chinese Biomedicine Database) (1978–2011.6) to identify cohort studies assessing the association between uric acid level and kidney allograft. Two authors independently screened the studies, assessed the risk of bias of included studies and extracted data. Unadjusted odds ratio(OR), mean difference (MD), adjusted hazard ratio (aHR) and their corresponding 95%CI were pooled to assess the effects of hyperuricemia on kidney allograft.

**Results:**

Twelve cohort studies were included and the quality was moderate to high based on the NEWCASTLE-OTTAWA quality assessment scale. RTRs with hyperuricemia had lower eGFR (P<0.0001, 95%CI−16.34∼6.14) and higher SCr (P<0.00001, 95%CI 0.17∼0.31) than those with normal uric acid level. Meta-analysis showed that hyperuricemia was a risk factor of chronic allograft nephropathy (Unadjusted OR = 2.85, 95%CI 1.84∼4.38, adjusted HR = 1.65, 95%CI 1.02∼2.65) and graft loss (Unadjusted OR = 2.29, 95%CI 1.55∼3.39; adjusted HR = 2.01, 95%CI 1.39∼2.94).

**Conclusions:**

Current evidence suggests that hyperuricemia may be an independent risk factor of allograft dysfunction. Hyperuricemia may modestly increase the risk of poor outcomes of RTRs. Future research is needed to verify whether lowering uric acid level could improve the kidney function and prognosis of RTRs with hyperuricemia.

## Introduction

Kidney transplantation has become a routine procedure in patients with end-stage renal failure as the development of kidney transplant technique and medicine. However, shortage of donors, the long-term outcomes and morbidity of kidney transplant patients are still remained problems [1]. Multiple factors contribute to long-term function and survival of the allograft. One study has shown that all kidney transplant recipients (RTRs) inevitably have or will have chronic kidney disease (CKD) as a result of the development of chronic allograft nephropathy(CAN) [2].

Increasing experimental and epidemiological studies suggest that hyperuricemia may play a role in the progression of cardiovascular and renal diseases. UA induces endothelial cell dysfunction, decreases nitric oxide production [3], [4], [5], stimulates vascular smooth muscle cell proliferation [6], [7], activates renin-angiotensin system [8] and produces various inflammatory mediators [9], [10]. Studies have found an independent association between hyperuricemia and hypertension, coronary heart disease, ischemic stroke, chronic renal diseases and type 2 diabetes [11].

Hyperuricemia is common among kidney transplant recipients, especially those on cyclosporine-based immunosuppressive regimens [12]. The incidence of hyperuricemia in kidney transplant patients, which was ∼25% before the routine use of cyclosporine, has increased to >80% with the widespread use of cyclosporine [12]. Increased UA level has been shown to be predictive of incident kidney disease and end-stage renal disease in those with normal renal function and progression of disease in individuals with kidney disease [13], while reduction of serum UA with allopurinol has been associated with slowing of the progression of renal disease [14]. Therefore, it has been proposed that hyperuricemia may have clinical significance in RTRs, but studies show conflicting results of the role of hyperuricemia in renal allograft recipients. We conducted a systematic review and meta-analysis of cohort studies to assess the association between serum UA levels and graft function and survival after kidney transplantation in an effort to clarify whether early-onset hyperuricemia is an independent predictor of long-term graft outcomes.

## Methods

### Searching

A comprehensive literature search was performed using databases MEDLINE (Ovid) from1948 until June 2011, Medline (R) in-process & other no-indexed citations (2011.5), EMBASE from 1956 until June 2011 and CBM (Chinese Biomedicine Database)from1978 until June 2011. The Medical Subject Heading (MeSH) ‘urate’, ‘uric acid’, ‘hyperuric’, ‘hyperuricemia’, ‘gout’, ‘transplant’ , ‘transplantation’ and ‘graft’ were used as English and corresponding Chinese search terms to identify studies from above database. The search strategies were adjusted based on the characteristics of each database. In addition, we searched the reference lists of all identified relevant studies.

### Selection

We only included prospective or retrospective cohort studies investigating the association of hyperuricemia with RTRs’ kidney function and long-term outcomes. Eligible studies must meet following criteria: patients with age older than 18, maintained intact renal function more than 6 months to minimize the effects of decreased graft function on hyperuricemia, and only one kidney transplanted. Patients were excluded if they had a history of malignant tumor, acute inflammation, acute renal allograft rejection and active liver disease.

Studies on drug trials, not published in English or Chinese, letters and review articles, and studies without available data were excluded.

### Validity Assessment

Two reviewers rated the quality of the eligible studies independently. Study quality was judged by using the NEWCASTLE-OTTAWA quality assessment scale. We assessed included studies based on three aspects: the selection of the study groups (0–4 points), the comparability of the groups (0–2 points), and the ascertainment of either the exposure or outcome of interest (0–3 points). The total score was 9. In case of disagreement, consensus was achieved by discussion with a third adjudicator.

### Data Abstraction

Data were extracted by two reviewers independently. For each study, we extracted information on author, publication year, country, study design, sample size, age and sex of RTRs, the definition of hyperuricemia, the length of follow-up and adjusted factors. Estimated glomerular filtration rate (eGFR) and serum creatinine (SCr) were as primary end-point, and CAN diagnosed by biopsy and graft loss during follow-up were as secondary end-point.

### Quantitative Data Synthesis

Extracted information was put into a table to help us browse the sample size, research design, quality and key features of each included study.

RevMan5.0 was used to pool unadjusted odds ratio(OR) of dichotomous variable, mean difference (MD) of continuous variable and obtain their corresponding 95%CI while STATA10 was used to pool adjusted hazard ratio (aHR) and obtain 95%CI. Chi-square test was used to assess the heterogeneity of included studies. We adopted a p-value of ≤0.1 as evidence of heterogeneity. The random effects model was used to pool data if there was heterogeneity, otherwise fixed effect model. A two-sided p value less than 0.05 was regarded as significant for all analyses. Subgroup analysis was performed to pool the effect size of prospective and retrospective cohort studies separately. Sensitivity analysis was performed by removing studies with distinctive definition of hyperuricemia. Meta-regression was performed to assess the effect of several clinical factors and study design on outcomes.

## Results

### Flow of Included Studies

**Figure 1:**
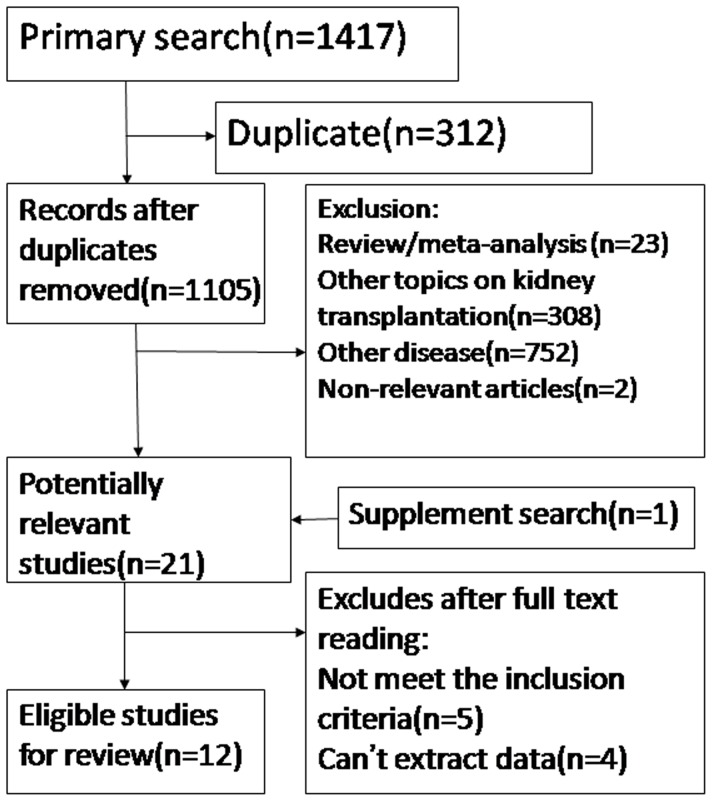
Flowchart of study selection.

The primary search strategy identified 1417 articles. After scanning the titles and abstracts, 1397 articles were excluded (reasons stated in Figure 1). After reading the full text of remaining studies and references tracing, 12 studies [15], [16], [17], [18], [19], [20], [21], [22], [23], [24], [25], [26] which met the inclusion criteria were included in the review (Figure 1).

### Study Characteristics and Quality

Table 1 summarized the characteristics of the included studies. Among these studies, six were prospective and six were retrospective cohort studies. The sample size ranged from 45 to 488. The studies were undertaken in U.S, China, Korea, Poland, German, Canadian, Turkey and Saudi Arabia. Eight studies [15], [17], [19], [21], [22], [23], [24], [25] with 2045 participants assessed the effects of UA on eGFR of RTRs; Five studies [18], [20], [21], [22], [23] with 873 participants assessed the effects of UA on SCr of RTRs; Five studies [19], [20], [21], [22], [24] with 1178 participants assessed the effects of UA on CAN and 5 studies [15], [16], [22], [24], [26] with 1519 participants assessed the effects of UA on graft loss (including death with functional graft). Follow-up time ranged from 1 to 10 years. All studies controlled the factors which had effects on kidney function more or less, including age, race, sex, diabetes, tumor, and medicines.

**Table 1:** The character of included study and score of quality assessment.

study	country	study design	sample size(H/N)	Age (H/N)	definition of hyperuricemia	Time of evaluating eGFR since transplant (month)	follow-up	comparable of baselines	adjusted factors	score of quality assessment
Haririan2011	U.S	Co,R	488	52.6±13.1	NS	12	41.1±17.7 months	Y	eGFR,race,donor,peak-PRA,HLA-mismatch,delayed graft function,acute cellular rejection,MMF dose,ACEI/ARB	8
Chung 2010	Korea	Co,P	148/208	H: 38.4±10.4N: 39±10.50	> 7.0 mg/dl for man;> 6.0 mg/dl for woman	12	N:105.68±31.9months;H:100.78±33.8 months	Y	age, sex, postoperative recovery pattern, the presence of diabetes or hypertension, BMI,retransplantation,donor type, HLA mismatch number, immunosuppressant type, and acute rejection episodes, TA-eGFR	9
Zou 2010	China	Co,R	58/84	NS	> 420 umol/l for man and > 380 umol/l for woman	12	34 for 1 year; 42 for 2 years;140 for 3 years	NS	unadjusted	7
			29/54			12				
Haririan 2010	U.S	Co,R	45/167	H: 50.5±12.9N: 47.2±13.9	> 7.0 mg/dL for man and>6.5 mg/dL for woman	12	68.3±27.2 months	Y	age, retransplantation,diabetes and induction	8
Karbowska 2009	Poland	Co,R	48/30	47.8/45.3	NS	NS	H:30.5 monthsN: 32.0 months	Y	unadjusted	8
Akalin 2008	Canada	Co,P	144/163	H: 50.0±1.0N: 47.7±1.0	> 7.0 mg/dL for man and >6.5 mg/dL for woman	6	mean 4.3 years	Y	age, race, sex, eGFR, having received a cadaveric transplant, and cyclosporin use.	8
Bandukwala 2009	Canada	Co,R	180/225	H: 50.2±11N: 50.3±12	>7.1 mg/dl for man and >6.1 mg/dl for woman	NS	N: 6.0±6 yearsH: 7.3±6years	Y	unadjusted	8
Kim 2010	Korea	Co,R	55/301	H: 36.8±10.8N: 39.7±10.3	>7.0 mg/dl for man and >6.0 mg/dl for woman	6	102.63±27.25months	Y	unadjusted	8
Min 2009	Korea	Co,P	24/97	H: 41.2±12.3N: 39.8±12.2	>8.0 mg/dl for both man and woman	12	∼5years	Y	unadjusted	8
Gerh0ardt U 1999	German	Co,P	80/268	41.1±12.78	> 8.1 mg/dL for man and > 6.1 mg/dL for woman	NS	5years	Y	unadjusted	9
Akgul2007	Turkey	Co,P	54/79	34.7±9.9	>7.0 mg/dl for man and >6.0 mg/dl for woman	NS	3years	NS	unadjusted	8
Abdelrahman2002	Saudi Arabia	Co,P	25/20	NS	>8.0 mg/dl for man and >6.0 mg/dl for woman	NS	8.9±4.6 years	Y	unadjusted	7

NS: not stated; N: normal uric acid group; H: hyperuricemia group; eGFR: estimated glomerular filtration rate; SCr: serum creatinine; Co: cohort study; R: retrospective; P: prospective; PRA: pane-reactive antibody.

We assessed the quality of included studies critically using NEWCASTLE – OTTAWA quality assessment scale. Two studies [16], [20] scored 9; two studies [17], [18] scored 7; the rest scored 8 (table1). The subjects of all included studies were patients with intact renal function for half a year after transplantation. Patients with hyperuricemia or normal serum uric acid level were comparable in baselines except 2 studies [17], [18] which did not report the relevant information. All studies except two [23], [26] described the definition of hyperuricemia. The follow-up of each study was more than 1 year and the drop-out was described.

### Hyperuricemia with eGFR

**Figure 2:**
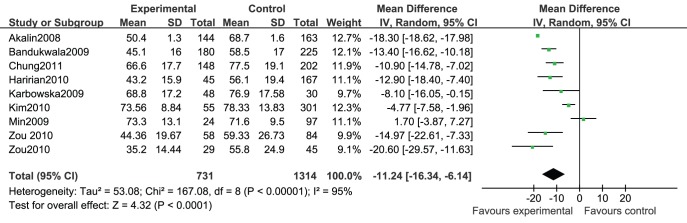
Association of hyperuricemia with eGFR of renal transplant recipients.

A total of 8 studies [15], [17], [19], [21], [22], [23], [24], [25] provided data on eGFR of RTRs with hyperuricemia versus normouricemia. There was significant heterogeneity among studies (*I^2^ = *95%, *p*<0.00001). Random-effects meta-analysis showed that RTRs with hyperuricemia had significantly lower eGFR than those with normal serum uric acid level (MD = −11.24, 95% CI −16.34∼−6.14) (Figure 2).

We performed a subgroup analysis by pooling prospective and retrospective studies separately and the results showed that there were significant heterogeneity among both prospective cohort studies (I^2^ = 93%, p<0.0001) and retrospective cohort studies (*I^2^* = 85%, *p*<0.00001). Random-effects meta-analysis showed that RTRs with hyperuricemia in both prospective and retrospective studies had significantly lower eGFR than those with normal serum uric acid level although the effect size of prospective cohort studies (MD = −14.86, 95% CI −22.10∼−7.63) was larger than that of retrospective cohort studies (MD = −9.99, 95% CI −15.00∼−4.98) (Figure 2).

Sensitivity analysis was performed by removing 3 studies [22], [23], [24] with distinctive definition of hyperuricemia, there was significant heterogeneity among studies (*I^2^ = *95%, *p* <0.00001). Random-effects meta-analysis still showed that RTRs with hyperuricemia had significantly lower eGFR than those with normal serum uric acid level (MD = −13.48, 95% CI −19.15∼−7. 81).

A random model meta-regression analysis was performed using the indicators of follow-up, race, definition of hyperuricemia, study type (*P* = 0.0016). This led to a relative decrease in heterogeneity (*R^2^* = 100%) with the residual of 0.00%. Meta-regression analysis disclosed significant interactions between race (*β* = −8.492514, *P* = 0.002), study type (*β* = 5.035018, *P = *0.013), definition of hyperuricemia (*β* = 10.0255, *P* = 0.006), and follow**-**up (*β* = 13.37142, *P* = 0.002).

### Hyperuricemia with SCr

We pooled the data of SCr of RTRs with hyperuricemia versus normouricemia in 5 studies [18], [20], [21], [22], [23]. There was no heterogeneity among these studies (*I^2^ = 44%, P = 0.13)*. Fixed-effect model meta-analysis showed that RTRs with hyperuricemia had significant higher SCr than those with normal serum uric acid level (MD = 0.24, 95%CI 0.17∼0.31) (Figure 3).

**Figure:**
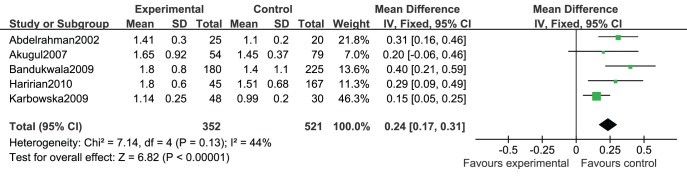
Association of hyperuricemia with SCr of renal transplant recipients.

### Hyperuricemia with CAN

**Figure 4:**
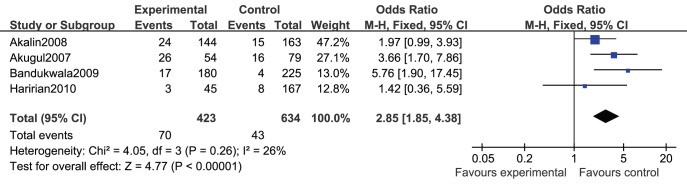
Relative risk of hyperuricemia with chronic allograft nephropathy.

The number of patients who developed CAN during the follow-up was available in 4 studies [19], [20], [21], [22]. There was no statistical significant heterogeneity among these studies (*I^2^* = 26%, *p* = 0.26*)*. Fixed-effect model meta-analysis showed that RTRs with hyperuricemia had significantly higher risk of developing CAN than those with normal serum uric acid level (unadjusted OR = 2.85, 95%CI 1.85∼4.38) (Figure 4).

Two studies [19], [24] reported the adjusted hazard ratio (aHR) of hyperuricemia with CAN of RTRs. There was no heterogeneity among the studies (*I* ^2^ = 0.0%, *P* = 0.444). Fixed-effect model meta-analysis showed that hyperuricemia was an independent risk factor of CAN of RTRs (aHR = 1.65, 95%CI 1.02∼2.65) (Table 2).

**Table 2:** Adjusted hazard ratio of hyperuricemia with graft loss, chronic allograft nephropathy (CAN).

Outcome	included study	Variable type	aHR (95%CI)	heterogeneity	meta analysis
				I^2^, P-value	aHR (95%CI)
hyperuricemia with graft loss	Min 2009	DV	2.01(1.09–3.72)	0.00%, 0.949	2.01 (1.39–2.94)
	Haririan2010	DV	1.92(1.1–3.4)		
	Chung2010	DV	2.3(0.9–5.8)		
hyperuricemia with CAN	Akalin2008	DV	1.28(0.57–2.84)	0.00%, 0.444	1.65 (1.02–2.65)
	Min 2009	DV	1.89(1.05–3.43)		
1 mg/dl increase of UA with graft loss	Haririan2010	CV	1.26(1.03–1.53)	0.00%, 0.649	1.21 (1.08–1.37)
	Haririan2011	CV	1.14(0.95–1.36)		
	Chung2010	CV	1.30(1.0–1.7)		

*DV: dichotomous variable; CV: continuous variable; aHR:adjusted hards ratio.*

### Hyperuricemia with Graft Loss

**Figure 5:**
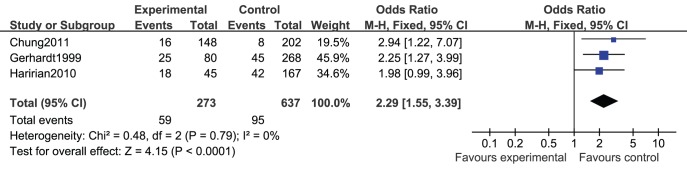
Unadjusted odds ratio (OR) of hyperuricemia with graft loss.

Three studies [15], [16], [22] reported the number of patients who suffered from graft loss. There was no heterogeneity among the studies(*I* ^2^ = 0%, *P* = 0.79). Fixed-effect model meta-analysis showed that RTRs with hyperuricemia had significantly higher risk of graft loss than those with normal serum uric acid level (unadjusted OR = 2.29, 95%CI 1.55∼3.39) (Figure 5).

Three studies [15], [22], [24] reported the aHR of hyperuricemia with graft loss of RTRs. There was no heterogeneity among the studies(*I* ^2^ = 0.0%, *P* = 0.949). Fixed-effect model meta-analysis showed that hyperuricemia was an independent risk factor of graft loss of RTRs (aHR = 2.01, 95%CI 1.39∼2.94) (Table 2).

Three studies [15], [22], [26] reported the aHR of increase 1 mg/dl uric acid with graft loss of RTRs. There was no heterogeneity among the studies (*I* ^2^ = 0.0%, *P* = 0.649). Fixed-effect model meta-analysis showed that uric acid was positively associated with graft loss of RTRs independently (aHR = 1.21, 95%CI 1.08∼1.37) (Table 2).

## Discussion

### Relationship of Hyperuricemia with Kidney Function of RTRs

Studies reported that UA may induce graft dysfunction, chronic allograft nephropathy and accelerate the progress of the chronic kidney disease through triggering inflammation, affecting vascular endothelial function and inducing microvascular injury. Intracellular uric acid could trigger oxidative stress reaction; activate inflammatory mediators such as interleukin-6, tumor necrosis factor-α, C-reactive protein [3], [4], [9], [10] and renin-angiotensin systems that finally lead to vascular endothelial dysfunction and microvascular injury [27], [28].

Our review found that eGFR in the hyperuricemia group was significantly lower than that in normouricemia group while the SCr level in the hyperuricemia group was significantly higher than that in normouricemia group. Both unadjusted and adjusted pooled effect size showed that the incidence rate of CAN and graft loss in hyperuricemia group was significantly higher than that in the normouricemia group, implying that hyperuricemia may be an independent risk factor of graft dysfunction and could modestly increase the risk of poor outcomes of kidney transplant receptors.

Clinical trials about the effects of urate-lowering therapy on kidney transplant recipients can provide direct evidence that higher uric acid is an independent risk factor of poor prognosis of renal transplant recipients. RCT performed by Goicoechea et al suggested that urate-lowering therapy slowed down renal disease progression of patients with chronic kidney disease (In the control group, eGFR decreased 3.3 ± 1.2 ml/min per 1.73 m(2), and in the allopurinol group, eGFR increased 1.3± 1.3 ml/min per 1.73 m(2) after 24 months) [29]. However, there are only 2 trials [30], [31] reported the effects of urate-lowering therapy on kidney transplant patients. Navascues et al reported that it is safe to use allopurinol to low hyperuricemia of kidney transplant recipients, but the SCr didn’t change significantly after treatment(2.35±0.92 mg/dl vs. 2.39±1.03 mg/dl) [31]. Flury et al compared the effects of allopurinol vs. benzbromarone in the treatment of hyperuricemia after kidney transplantation, both drugs effectively lowered serum uric acid concentrations by 19% vs. 35% of pretreatment values [30]. Unfortunately, there is no evidence to suggest that the allograft function of patients with urate-lowering therapy is better than that of kidney transplant recipients without urate-lowering drug. Therefore, high quality evidence is needed to show that decreased uric acid level is related to good prognosis of kidney transplant recipients.

### Hyperuricemia and Graft Dysfunction, Cause or Effects?

Kidney is an important metabolic pathway of uric acid. When kidney function is compromised, the excretion of uric acid decreases while the level of serum uric acid increases. One study reported that renal dysfunction is associated with higher UA levels, but higher UA levels is not parallel with the progression of renal dysfunction independently after kidney transplantation [32]. Min et al found that recipients in the early-onset hyperuricemia (within 1 year after transplantation) had a stable graft function. The eGFR are 58.7±10.8, 58.9±11.5 and 58.8±10.7 ml/min/1.73 m^2^, respectively at detection of hyperuricemia, at 6 months post-detection, and at 12 months post-detection [24]. Chung et al found that UA-level of RTRs increased from 5.3±1.9 mg/dl to 6.3±1.7 mg/dl within 3 months post-transplant, but the eGFR didn’t change significantly. The 1-year eGFR was significantly lower in the hyperuricemic group (68.3 8± 20.4 ml/min/1.73 m^2^) than that in the normouricemic group (77.9 8±19.2 ml/min/1.73 m^2^) (p = 0.000) [15]. Haririan et al found that RTRs with hyperuricemia during early post transplant period had significantly higher 1-year SCr, lower eGFR, and worse graft survival when compared with those with normal mean 6-month UA values [22]. The results of above studies in combination with our meta-analysis indicated that hyperuricemia may be an independent risk factor of graft dysfunction and may affect the life span of recipients of kidney transplantation.

### Dose-response Association of Uric Acid Level with Graft Function

Min et al found that early-onset moderate-to-severe hyperuricemia (defined as UA≥8.0 mg/dl) was found to be a significant risk factor for CAN (P = 0.035) and a poorer graft survival (P = 0.026) by multivariate analysis, but not mild hyperuricemia. In moderate-to-severe hyperuricemia group, the mean eGFRMDRD (Modification of Diet in Renal Disease) at the fourth post-transplant year were significantly lower than those at 1 year post-transplant(53.7±15.2 vs. 61.9±12.5). The impact of hyperuricemia on RTRs was dependent on the duration of exposure. Likewise, the detrimental effect of early-onset hyperuricemia on the graft function was dependent on UA-level and exposure time. After control of the baseline graft function by analysis of only recipients with a good graft function at 1 year post-transplantation (eGFR > 60 ml/min), moderate-to-severe early-onset hyperuricemia was also a marker of long-term graft dysfunction and failure [24]. Haririan et al found that higher UA level had significant higher risk of graft loss compared to lower UA level. Each 1 mg/dL increase of UA level was associated with a 15% increase on average in the risk of graft loss during the study period and seemed to be associated with increased risk of death [26]. These two studies suggested that both higher UA-level and longer duration of exposure have severe impacts on graft function.

### Advantages and Limitations

This meta-analysis has some advantages. First, the samples of included cohort studies came from Korea, China, Canada, Poland, Saudi Arabia, Turkey, USA and German that would have a good representative of race. Second, the effect of Hyperuricemia on renal function is consistent regardless of whether other confounding factors were adjusted or not. Third, the included studies have moderate to high quality and good internal validity.

Limitations of this study should be noted. First, the included studies had potential risk of bias due to differences in complete reporting of follow-up and judgment of outcomes. Second, although included study adjusted confounding factors relevant to renal function more or less, the number and types of adjusted factors were different. There are maybe residual confounding factors which have effects on outcomes. However, the pooled estimates found that no matter confounding factors adjusted or un-adjusted, no matter how many confounding factors were adjusted, the conclusion that hyperuricemia is a risk factor of graft dysfunction and poor prognosis is consistent. Third, as the sample size of this meta-analysis was small, we can’t get the definitive conclusions. Fourth, our review only included studies which have been published in English or Chinese and did not search grey literature, publication bias may exist. We could not assess the publication bias precisely due to the limited number of included studies for each outcome (<10).

### Conclusions and Implications for Clinical Practice and Research

Our systematic review shows that hyperuricemia has effects on eGFR and SCr of RTRs and hyperuricemia may be an independent risk factor of CAN and graft loss. Because this review is based on observational studies, the number of included studies and the sample size are limited and uric acid lowering drugs have potential bad effects on kidney function, the application of uric acid lowering drugs for RTRs with hyperuricemia is not yet routinely recommended. Future cohort studies need pay more attention to the representative of study people, the comparable baselines of different groups, the adjustment of confounding factors, long-term follow-up and assessment of outcomes. High quality clinical trials are needed to verify whether reducing the serum uric acid level can delay the kidney dysfunction after kidney transplantation, the process of chronic allograft nephropathy and the death of RTRs, and to provide basis for the reasonable application of uric acid lowering drugs.
